# Crosstalk Between Astrocytes and Inhibitory Neurons During Maturation: Emerging Mechanisms and Functional Implications

**DOI:** 10.1111/jnc.70266

**Published:** 2025-11-06

**Authors:** Niina Lehti Tauriala, Vered Kellner

**Affiliations:** ^1^ Department of Neurophysiology and Neuropharmacology, Centre for Physiology and Pharmacology Medical University of Vienna Vienna Austria

**Keywords:** activity‐dependent, astrocytes, development, inhibitory neurons

## Abstract

Astrocytes, once regarded merely as passive support cells in the brain, have recently emerged as active partners to neurons in sensory processing, learning and memory. By promoting the development, maturation and refinement of neuronal synapses, astrocytes play a central role in shaping brain circuitry. Within these circuits, inhibitory neurons comprise approximately 20% of brain cells, with variation across regions and developmental stages. The fundamental importance of inhibition is underscored by its evolutionary conservation, being present even in primitive nervous systems. Notably, astrocyte development coincides temporally with a peak in synaptogenesis and with the maturation of inhibitory neurons, suggesting potential interplay between these processes. Historically, research has primarily focused on astrocyte interactions with excitatory neurons; however, there is growing recognition of the likely significance of astrocyte–inhibitory neuron crosstalk, particularly during critical developmental periods. Here we review current knowledge on the development of astrocytes and inhibitory neurons, highlight emerging evidence for their interactions and propose hypotheses to guide future research.

## Graphical abstract

In this review, we discuss the development and maturation of inhibitory neurons and astrocytes and how early developmental activity in the brain might promote the interactions between these cell types. Early in development, excitatory neurons are the first to emerge. As they migrate and mature into their set places, glutamate is released into the extracellular environment. Precursors for inhibitory neurons which will later become parvalbumin (PV+) or somatostatin (SST+) neurons begin to emerge and migrate. Once astrocytes emerge, they respond to glutamate release from excitatory neurons with increases in calcium. Our hypothesis is that this enables inhibitory neurons to mature. Future research is needed to understand these complex reciprocal interactions that promote a healthy and mature brain.

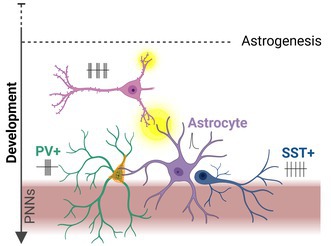

**Abbreviations**
5HT3a: 5‐hydroxytryptamine type 3A
AMPA: α‐Amino‐3‐hydroxy‐5‐methyl‐4‐isoxazolepropionic acid
BDNF: Brain‐derived neurotrophic factor
BrdU: 5‐bromo‐2‐deoxyuridine
CA1: Cornu ammonis 1
CB: Calbindin
CB1: Cannabinoid receptor 1
CCK: Cholecystokinin
CCN1: Cellular communication network factor 1
E/I: Excitation/inhibition
ECM: Extracellular matrix
Eph: Ephrin
Fgfr: Fibroblast growth factor receptor
FXS: Fragile‐X syndrome
GABA: γ‐aminobutyric acid
GABAa: GABA Type A receptor
GABAb: GABA Type B receptor
GAT: GABA transporter
Glua2: Glutamate ionotropic receptor AMPA type subunit 2
HAPLN1: Hyaluronan and proteoglycan link protein 1
IPSCs: Inhibitory postsynaptic currents
KCC2: Potassium chloride cotransporter 2
Mecp2: Methyl CpG binding protein 2
Megf10: Multiple EGF‐like domains 10
Mertk: MER proto‐oncogene tyrosine kinase
MGE: Medial ganglionic eminence
mIPSCs: Miniature inhibitory postsynaptic currents
MMP: Matrix metalloproteinase
NKCC1: Sodium–potassium chloride cotransporter 1
NPY: Neuropeptide Y
NrCAM: Neuronal cell adhesion molecule
NT: Neurotrophin
P2X: Purinergic type 2X receptor
P2Y: Purinergic type 2Y receptor
PNN: Perineuronal net
PV: Parvalbumin
sIPSCs: Spontaneous inhibitory postsynaptic currents
SPARC: Secreted protein acidic and rich in cysteine
SST: Somatostatin
TrkB.T1: Tropomyosin receptor kinase B, truncated isoform 1
VIP: Vasoactive intestinal peptide

## 1 Inhibitory Neuron Development and Maturation

A mature and healthy brain requires various levels of specialised inhibition to ensure proper functioning. For this reason, inhibitory neurons have evolved into a vastly heterogeneous collection of subtypes that vary according to their morphology, cellular content, function and secreted products. In the mature brain, the three major classes of inhibitory neurons: parvalbumin‐expressing (PV+), somatostatin‐expressing (SST+) and vasoactive intestinal peptide‐expressing (VIP+) neurons, are largely distinct and nonoverlapping, though additional subclasses also exist, often sharing overlapping molecular markers (Markram et al. 2004; Pfeffer et al. 2013; Taniguchi et al. 2013). It should be noted that most of the research on inhibitory neuron development has focused on the cortex, but in other brain areas, the three main classes mentioned above are not always purely inhibitory and the timing of their appearance can vary (see reviews on other brain regions: Benevento et al. 2022; Li et al. 2014; Schilling 2024; Liu et al. 2024).

Of the most well‐studied, PV+ inhibitory neurons provide fast, perisomatic and highly accurate inhibition critical for synchronising neuronal circuits and amplifying network signals, thus exerting global yet fine‐tuned inhibitory control (Celio 1986; Lee et al. 2016; Runyan et al. 2010). In contrast, SST+ inhibitory neurons are regular‐spiking cells that target apical dendrites of pyramidal neurons, allowing them to deliver a more generalised, slower and steadier state of inhibition and hence are often associated with top‐down modulation and the maintenance of sensory integration and plasticity (Dai and Sun 2025; Gibson et al. 1999; Urban‐Ciecko and Barth 2016). SST+ neurons selectively avoid connections with other SST+ neurons, but will connect with both excitatory and inhibitory neurons, often in a layer‐specific manner (Pfeffer et al. 2013; Wu et al. 2023; Xu et al. 2013). VIP+ neurons primarily act upon other inhibitory neurons, particularly on SST+ neurons, allowing for a disinhibitory effect on excitatory neurons (Millman et al. 2020; Myers‐Joseph et al. 2024; Piet et al. 2024). The variations in function among inhibitory neurons are the key to developing a well‐balanced, mature brain. Although the value of further subdividing each class of inhibitory neuron remains a matter of intense debate in the field, our discussion will focus on the broad categories outlined above (for further reading about inhibitory neuron subtypes: Helm et al. 2013; Park et al. 2025).

### 1.1 Distinctive Dynamics of Inhibitory Neuron Maturation

The maturation of inhibitory neurons exhibits several features that set them apart from other neuronal populations. Unlike excitatory neurons, whose lineage is a strong predictor of final positioning, the ultimate location of inhibitory neurons in the mature forebrain cannot be reliably inferred from lineage tracing alone. After their generation, inhibitory neurons undergo extensive dispersion (Mayer et al. 2016). Whereas excitatory neurons migrate radially in a characteristic inside‐out pattern, inhibitory neurons follow tangential migratory routes that run parallel to the brain's surface. These widespread and distinctive migration patterns suggest that factors beyond lineage, such as local environmental signals and cell–cell interactions, play a central role in guiding their final distribution (Harwell et al. 2015; Inada et al. 2011; Miyoshi et al. 2015). Another key distinction is temporal: inhibitory neurons integrate into cortical circuits only after excitatory neurons have populated their target areas, beginning around embryonic day 10 (E10) in the mouse (Angevine and Sidman 1961). Moreover, inhibitory neurons do not express their characteristic molecular markers until postnatal stages (Wamsley and Fishell 2017), though it was shown that their differentiation into subclasses can be distinguished already at the progenitor cell level (Bandler et al. 2022). The precise mechanisms for this remain incompletely understood; however, genetic and transcriptional programs are known to influence their differentiation (see Lim et al. 2018 for a review). Excitatory neurons can also promote inhibitory neuron migration and specialisation within their vicinity (Cadilhac et al. 2021; Lodato et al. 2011; Wester et al. 2019).

Inhibitory neurons originate from distinct regions of the ventral forebrain rather than from the dorsal pallium, which gives rise to excitatory neurons (Anderson et al. 1997; Marín and Rubenstein 2001; Wonders and Anderson 2006). The medial ganglionic eminence (MGE) generates most inhibitory neurons, including PV+ and SST+ classes and their respective subclasses. The caudal ganglionic eminence (CGE) contributes additional populations, notably the VIP+, cholecystokinin (CCK+) and Neurogliaform‐type neurons (Butt et al. 2005; Horton and Paredes 2025; Miyoshi et al. 2010; Warm et al. 2022). Notable contributions also arise from the preoptic area and adjacent subpallial zones (Gelman et al. 2009; Wonders and Anderson 2006). CCK+ and neuropeptide Y (NPY+) expressing inhibitory neurons exhibit overlap with PV+ or SST+ neuron markers, expanding the array of functions they have, but are out of the scope of this review (Gonchar et al. 2008; Grieco et al. 2023).

### 1.2 Staggered Development of MGE‐Derived Inhibitory Neurons

**FIGURE 1:**
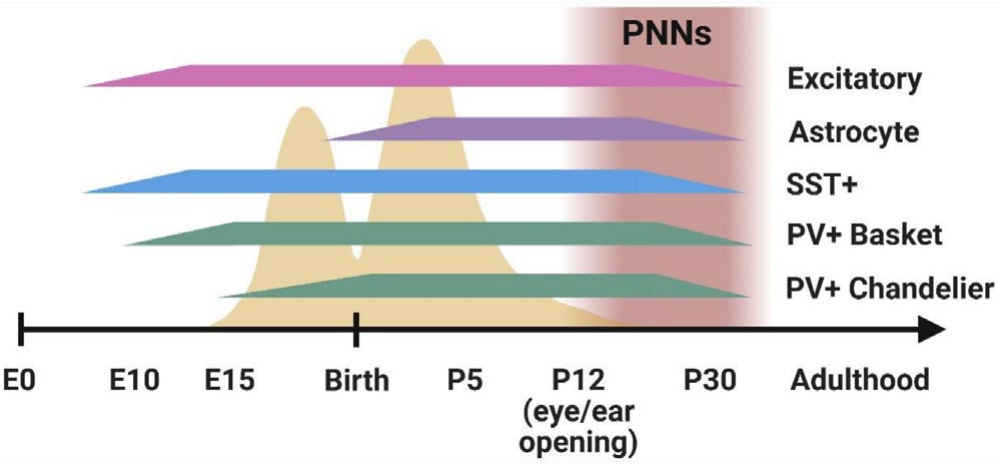
Developmental timeline of inhibitory neurons and astrocytes in the developing mouse brain. Synaptogenesis occurs in two peaks during development, once before birth and secondly after birth (yellow peaks). Perineuronal net (PNN) formation begins around the time of eye and ear opening in mice (Postnatal day 12; P12) and continues until the fourth week of life. Bars represent the current understanding of the emergence of each cell type. Expression of classical cell‐specific markers (i.e., somatostatin (SST+), parvalbumin (PV+)) is present after eye opening. Most cell types are considered matured by P30. Excitatory neurons emerge from progenitor cells around embryonic day 10 (E10) and are produced until all cortical layers are complete (Angevine 1965). Astrocytes emerge immediately before birth around E18 (Morrow et al. 2001) and mature postnatally around the time of eye opening; full maturation extends to the end of the fourth week of life (Tabata 2015). Somatostatin neurons emerge and mature from medial ganglionic eminence (MGE) derived precursor cells prior to PV+ neurons and mature by the end of the second postnatal week (P12–14), but continue developing synapses up to the fourth week (Pan et al. 2016). Note that no studies were found that directly show a birthdate for SST+ neurons; studies use the first appearance of MGE‐derived neurons to represent SST+ neurons emergence. Basket cells appear between E9.5 to E15.5 (Caroni 2015) and complete functional maturation postnatally as PNNs establish around their soma. Chandelier cells appear slightly after, around E13.5–17 (Taniguchi et al. 2013), and also mature postnatally as PNNs enwrap their soma. Studies used either lineage tracing or BrdU‐based (5‐bromo‐2‐deoxyuridine) birth dating to identify birthdates of cell types. Ranges of birthdates within a neuronal subclass likely exist due to the further subdivisions within the subclasses.

There is a developmental sequence among the different subclasses of inhibitory neurons, where earlier‐born neurons play a crucial and distinct role in shaping the growth and integration of the later‐born neurons (Figure 1) (Hosseini Fin et al. 2025; Mayer et al. 2018; Mòdol et al. 2024).

SST+ neurons are the first to emerge in the developing brain and have been shown to control the appearance of succeeding PV+ neurons (Jiang et al. 2025; Lazarus and Huang 2011; Miyoshi and Fishell 2011; Tuncdemir et al. 2016). Previous research revealed two clear functions of SST+ neurons: firstly, to establish large generalised and synchronised circuit‐wide inhibitory control of early networks via giant depolarising potentials, and secondly, to prevent precocious PV+ neuron maturation (Mohajerani and Cherubini 2006; Tuncdemir et al. 2016). When SST+ neurons are inhibited, PV+ neurons mature earlier. As development progresses, PV+ neurons influence the shift of neuronal activity to a sparser and less synchronised state characteristic of a mature brain. Thus, this shift from synchronised to asynchronous activity is controlled by SST+ neurons and marks the transfer of precise inhibitory regulation from SST+ to PV+ neurons, leading to the formation of early coordinating inhibitory networks in mature circuits (Mòdol et al. 2024).

PV+ neurons themselves are not uniform, but are divided into PV+ basket or chandelier cells (Jones 1975; Miyamae et al. 2017; Szentágothai 1975). Basket cells have horizontal axonal arborisation patterns which target the soma and proximal dendrites of pyramidal neurons, while chandelier cells target the axon initial segments and control action potential initiation (DeFelipe et al. 1986, 1989; Gallo et al. 2020; Palay and Chan‐Palay 2012; Ramón y Cajal 1909). Both basket and chandelier cells appear before birth but mature postnatally after the second peak of synaptogenesis (Wang et al. 2002). Basket cell precursors first appear around E9.5 (Donato et al. 2015), followed by chandelier cell precursors around E13.5–17 (Gallo et al. 2020; Taniguchi et al. 2013). When basket cells reach their target area, they establish large perisomatic synapses onto pyramidal neurons to initiate broad inhibitory control of the forming circuitry to stabilise network activity and prevent hyperexcitability (Cobb et al. 1995). As these establish, chandelier cells complete their migration and begin to mature, selectively targeting axon initial segments of pyramidal neurons to provide precise, fine‐tuned inhibitory modulation in the now more mature circuit (P13.5–15 in mice; Inan et al. 2012). Around the peripubertal stage (P30 in mice), both cell types possess their hallmark fast‐spiking phenotypes and continue maturation into early adulthood (Miyamae et al. 2017). Similar subdivisions within SST+ neurons with staggered developmental emergence have also been shown (Fisher et al. 2024). Sequential developmental timelines of inhibitory neuron subtypes therefore ensure a carefully orchestrated transition from an early excitatory brain to a stable and finely tuned mature system.

### 1.3 Perineural Nets

A key feature of inhibitory neuron maturation is the formation of the well‐studied and specialised extracellular matrix (ECM) structures known as perineuronal nets (PNNs), which envelop the soma and proximal dendrites of fast‐spiking PV+ neurons (Härtig et al. 1992; Santos‐Silva et al. 2024). Once considered static structures, PNNs are now recognised as dynamic assemblies composed of multiple unique protein aggregates of the likes of chondroitin sulfate proteoglycans (CSPGs; including aggrecan, brevican, neurocan, phosphacan, neuroglycan‐C and versican) along with hyaluronan, tenascin‐R and various linking proteins (Giamanco and Matthews 2012; Sigal et al. 2019). PNN appearance in the developing brain has been linked to the closure of critical periods, marking the beginning of mature circuit establishment (Carulli and Verhaagen 2021; Mirzadeh et al. 2019; Pizzorusso et al. 2002). They have also been associated with ionic homeostasis and protection from oxidative stress (Morawski et al. 2004; Tewari et al. 2024). PNNs form a controlled microenvironment of synaptic exchange by helping guide neurotransmitters and ions through holes in the ECM that are contacted by astrocyte processes for rapid recycling and clearance. This highlights PNNs as an essential structural and functional component of the tripartite synapse (Araque et al. 1999). Without PNNs, astrocytes extend abnormally over the soma of inhibitory neurons, disrupting normal synaptic functioning and leading to seizures in mice (Tewari et al. 2024).

Sex differences in PNN maturation arise during the period in which PV+ neurons mature, during puberty (Drzewiecki et al. 2020; Griffiths et al. 2019; Meyer et al. 2014). The increase in Estrogen is thought to limit the maturation of PNNs in females, with Er‐α receptors that colocalise intrinsically with PV+ neurons likely playing a role (Hernández‐Vivanco et al. 2025; Wu et al. 2014). When the timely development of PNNs around inhibitory neurons is disrupted, neurological disorders with a sex bias are likelier to arise (de Carvalho et al. 2023; Jakovljevic et al. 2022; Rahmani et al. 2023; Wen, Afroz, et al. 2018; Wen, Binder, et al. 2018; Woodward and Coutellier 2021; Zhang et al. 2021).

### 1.4 Activity Within Inhibitory Circuits

As with excitatory neurons, inhibitory neurons undergo programmed cell death in the absence of electrical activity (Denaxa et al. 2018). Wong et al. (2018) demonstrated that pyramidal neuron spontaneous activity in the cortex of P7–8 mice determines the survival of inhibitory neurons via PTEN signalling. Notably, inhibitory neurons with the most spontaneous activity were also the ones likeliest to survive into adulthood. In parallel, Priya et al. (2018) found that the calcium‐dependent protein phosphatase, calcineurin, was critical for inhibitory neuron survival. During the earliest stages of inhibitory development, giant depolarising potentials, driven by excitatory GABA signalling, are believed to generate the first robust waves of activity within inhibitory neurons, thus promoting the formation and maturation of inhibitory circuits (Allene et al. 2012; Cherubini et al. 2011; Mohajerani and Cherubini 2006). Reduced GABA release onto pyramidal neurons leads to excessive network synchrony, thereby limiting the required apoptosis of MGE‐derived inhibitory neurons necessary to stabilise the developing cortical network (Duan et al. 2020).

Inhibitory neuron spontaneous activity plays a crucial role in shaping their circuitry. Basket cells in the dentate gyrus receive excitatory input from cortical neurons, and this in turn influences their morphology and further enhances circuit development (Pieraut et al. 2014). Spontaneous activity of SST+ neurons plays a pivotal role in controlling maturation and integration of PV+ neurons (Mòdol et al. 2024). Once PV+ neurons are established, they restrain the effects of the earlier appearing SST+ neurons on synaptic refinement (Jiang et al. 2025). This reciprocal and antagonistic relationship between the two cell types ensures excessive synapses are pruned, supporting stable electrical activity in the maturing brain. SST+ neurons also generate vital extracellular products for PV+ neuron synaptogenesis, such as Collagen XIX (Su, Basso, et al. 2020; Su et al. 2016). Another class of inhibitory neuron that expresses 5HT3a serotonin receptor was also shown to sharpen sensory maps and memory through early postnatal spontaneous activity at P6 (Che et al. 2018). It is evident, therefore, that spontaneous bursts of activity within developing inhibitory networks are vital for the establishment of mature circuitry.

Disruptions in inhibitory neuron activity have also been evidenced in models of neurodevelopmental disorders known to have E/I imbalances, such as in autism, schizophrenia or Fragile‐X Syndrome (FXS). Studies show that restoring the activity of pathologically hypoactive PV+ neurons after the critical period in FXS models (Kourdougli et al. 2023) or blocking the aberrant persistent connectivity between thalamocortical and SST+ neurons (Dumontier et al. 2024) rescues cognitive and behavioural deficits in mice by reinstating a healthy state of inhibition to the system. PNNs are also well known to be altered in multiple disorders (Wen, Binder, et al. 2018). In rodent models where PNNs were disrupted, PV+ neuron activity was significantly reduced, and the circuit returned to a more juvenile state by displaying heightened plasticity (Lensjø et al. 2017). Specific proteins within the PNN critically regulate PV+ neuron plasticity, often in an activity‐dependent manner (e.g., Favuzzi et al. 2017). When PNNs are disrupted, behavioural symptoms arise that are typical of imbalances in the E/I activity in neurodevelopmental disorders.

## 2 Astrocyte Development and Maturation

Astrocytes originate during late embryonic stages from radial glia, guided by a combination of intrinsic and extrinsic signals (Clavreul et al. 2019). Following their specification, astrocytes migrate to their target locations, though this migration is generally more limited compared to neurons or oligodendrocytes (Jacobsen and Miller 2003). Once positioned, they proliferate and continue maturing postnatally in rodents by elaborating their fine processes and establishing distinct, nonoverlapping domains (Bushong et al. 2004).

Traditionally, astrocytes have been categorised into fibrous and protoplasmic types based on their localisation within the white and grey matter, respectively (Andriezen 1893). However, astrocyte classification extends beyond these broad categories, encompassing more subtle distinctions that reflect their considerable heterogeneity (see Matias et al. 2019 for a comprehensive review). This heterogeneity extends to their developmental origins as well, with some astrocytes arising directly from radial glia while others proliferate from existing astrocytes (Clavreul et al. 2022) and others derive from a second type of radial glia to give rise to Olig2+ astrocytes (Zhou et al. 2025). Additionally, subsets of cortical astrocytes express sonic hedgehog receptors—key components of a signalling pathway involved in brain patterning—although the functional relevance of this expression remains to be elucidated (Garcia et al. 2010).

Astrocyte development is profoundly influenced by the activity of nearby neurons. During postnatal development, neuronal activity directly affects astrocyte growth and maturation through multiple pathways (Hasel et al. 2017). Experimental blockade of neuronal excitatory activity leads to reduced astrocyte morphological complexity, mediated by metabotropic glutamate receptor 5 (mGluR5; Morel et al. 2014), and induces transcriptomic changes within astrocytes (Farhy‐Tselnicker et al. 2021). The interplay between astrocytes and neurons has been further investigated in vivo, particularly within developing sensory circuits. A hallmark of early neural development is spontaneous burst firing by neurons, occurring prior to sensory experience and the maturation of stimulus‐driven activity (Feller 1999). Recent studies reveal that astrocytes also exhibit similar spontaneous activity patterns before auditory circuit maturation (Kellner et al. 2021). In Drosophila, astrocytes engage in coordinated activity with neurons during development (Akin et al. 2019); notably, while blocking astrocytic calcium signalling does not eliminate neuronal spontaneous activity, complete astrocyte ablation nearly eradicates it (Bajar et al. 2022). Collectively, these findings show that astrocytes and neurons interact during development to promote a mature, functioning circuit.

While extensive research has elucidated the role of excitatory neuronal activity in promoting astrocyte development, the influence of inhibitory neuronal activity on astrocyte maturation remains comparatively underexplored and warrants further investigation.

## 3 Astrocytes Can Sense GABA

Astrocytes express various transporters and receptors that enable them to detect and respond to the inhibitory neurotransmitter γ‐aminobutyric acid (GABA). The GABA transporters GAT1 and GAT3 are highly expressed by astrocytes in rodents starting from birth, with their expression levels increasing throughout development (Clarke et al. 2018; Martinez‐Lozada et al. 2023; Vitellaro‐Zuccarello et al. 2003). During the second and third postnatal weeks, these transporters shift in localisation from astrocyte cell bodies to their fine processes, highlighting developmental changes in GABA uptake mechanisms (Vitellaro‐Zuccarello et al. 2003). Activation of astrocytic GABA transporters triggers increases in intracellular calcium via sodium‐mediated calcium‐induced calcium release from internal stores (Boddum et al. 2016; Doengi et al. 2009).

In addition to GABA transporters, astrocytes express ionotropic GABAa and metabotropic GABAb receptors, both of which elicit calcium responses upon activation. GABAa receptor activation causes calcium influx directly from the extracellular space (Meier et al. 2008; Nilsson et al. 1993), whereas GABAb receptor‐mediated calcium increases depend on release from intracellular stores (Bostel et al. 2025; Covelo and Araque 2018; Kang et al. 1998; Mariotti et al. 2016, 2018; Nagai et al. 2019; Perea et al. 2016). The expression and functional responsiveness of GABAb receptors are developmentally regulated, peaking around P11 in the hippocampus (Meier et al. 2008), which suggests a role in brain maturation. Furthermore, astrocytes show increased mRNA expression during development for enzymes, receptors and proteins involved in GABA synthesis, release and uptake, with most peaking near P14—concurrent with the maturation timeline of inhibitory neurons (Ozkan and Koh 2026).

Early studies indicated that GABA influences astrocyte morphological complexity through GABAa receptor activation (Matsutani and Yamamoto 1997; Mong et al. 2002). More recently, Cheng et al. (2023) demonstrated that inhibitory neuron activity regulates astrocyte morphology in vivo during development via GABAb1 receptors on astrocytes. Overall, while it is clear that astrocytes are responsive to GABA, the precise physiological conditions under which astrocytes encounter and sense GABA during development remain to be elucidated.

## 4 The GABA Shift

In the late 1980s, Ben‐Ari et al. (1989) showed in neonatal rodent hippocampal slices that immature neurons respond to GABA with depolarisation, rather than the classical inhibition observed in mature neurons. This paradoxical excitatory action is attributed to a high intracellular chloride concentration maintained by the sodium–potassium chloride cotransporter NKCC1 during early development (Yamada et al. 2004). As development proceeds, the expression of the potassium chloride cotransporter KCC2 increases, facilitating chloride extrusion from neurons and lowering intracellular chloride levels (Rivera et al. 1999). This shift enables GABAergic signalling to switch from excitation to inhibition, a phenomenon termed the ‘GABA shift’. The precise timing and functional significance of this shift have been extensively studied, underlining its critical role in the maturation of neural circuits (Chancey et al. 2013; Deidda et al. 2015; Wang and Kriegstein 2011).

Despite robust evidence from brain slice preparations, the existence and timing of the GABA shift in vivo have generated considerable debate. Brain slice studies may alter chloride gradients, oxygen availability and energy substrates, potentially skewing the interpretation of network dynamics (Bregestovski and Bernard 2012; Zilberter 2016). For example, Kirmse et al. (2015) showed that while GABA depolarises individual neurons in the cortical plate at P3–4 in vivo, it exerts an inhibitory influence at the network level. Similarly, Valeeva et al. (2016) demonstrated a clear GABA switch in hippocampal and cortical slices around P9, yet observed predominantly inhibitory effects at the same age in living animals. More nuanced findings from Murata and Colonnese (2020) using chemogenetic and optogenetic methods in awake, unanaesthetised mouse pups revealed region‐ and age‐specific inhibitory neuron effects: inhibitory neurons excite CA1 pyramidal cells at P3 but become inhibitory by P7, while inhibitory neurons in the visual cortex appear inhibitory already at P3. These data suggest that GABA may not be universally depolarising in all brain regions in vivo during early postnatal stages.

If we assume that GABA *is* depolarising during development in the intact brain, a critical question arises: how is excessive excitation managed during development when both glutamate and GABA display excitatory actions? One proposed mechanism is shunting inhibition, where activation of GABAa receptors increases local membrane conductance, dampening excitability and reducing the impact of excitatory inputs without necessarily causing hyperpolarisation (Blaesse et al. 2009). Another possibility is that glutamate itself inhibits GABA release from inhibitory neurons, effectively reducing inhibitory output (van den Pol et al. 1998).

We propose a third mechanism involving astrocytes in regulating excess excitation during early development. Our previous work demonstrated that astrocytes in the inferior colliculus, the auditory centre of the midbrain, respond to intense synchronised neuronal events with robust calcium elevations mediated by metabotropic glutamate receptors mGluR5 and mGluR3 (Kellner et al. 2021). Given that astrocytic mGluR5 is highly expressed during early developmental periods (Cai et al. 2000; Sun et al. 2013) and closely linked to glutamate transporter expression and function (Devaraju et al. 2013; Morel et al. 2014; Umpierre et al. 2019), we hypothesise that astrocyte activity coordinates glutamate uptake to limit overexcitation until GABAergic signalling becomes inhibitory. GABA uptake might also be involved, but currently this remains unknown. Additionally, astrocytes may regulate neuronal excitability by releasing ATP as a gliotransmitter through calcium‐dependent exocytosis or membrane channels (see Illes et al. 2019 for a review). ATP acts on neuronal P2Y and P2X receptors to exert inhibitory effects, further contributing to reduced excitation (Bowser and Khakh 2004; Koizumi et al. 2003; Lalo et al. 2014; Zhang et al. 2003).

## 5 Coincident Timing of Astrocyte and Inhibitory Neuron Maturation

In the mouse brain, synaptogenesis peaks in two waves, the first immediately before birth and then again shortly after birth (Reemst et al. 2016). This second, postnatal surge in synapse formation coincides with the onset of astrogenesis (Freeman 2010) and has been shown in invertebrates to rely on astrocytes (Muthukumar et al. 2014), inciting the possibility of mammalian systems following a similar pattern. The close timing of astrogenesis and synaptogenesis is likely no accident, but instead reflects a precisely coordinated developmental program in which astrocytes are central to assembling and refining neural networks during a period of heightened plasticity.

The coordination of neuronal spontaneous activity and astrocyte calcium elevations is restricted to the developmental period and greatly reduces once neuronal activity changes to sparse, asynchronised firing (Kellner et al. 2021). Around this time point, precursor neurons begin to express parvalbumin in the brain (Alcántara and Ferrer 1994; del Rio et al. 1994) and begin their maturation, which will complete only after 4 weeks in rodents (Doischer et al. 2008; Goldberg et al. 2011) and after several years in humans (Fung et al. 2010; Rogers et al. 2018). The maturation of parvalbumin neurons involves the increased expression of the calcium binding protein parvalbumin and the establishment of PNNs around their cell bodies (Wintergerst et al. 1996). The proteoglycans of which PNNs are composed gradually appear around the second postnatal week in rodents. This activity‐dependent process allows for the gradual envelopment of PV+ neuron soma, with PNN establishment ending critical periods of development (Favuzzi et al. 2017; Nakayama et al. 2025; Pizzorusso et al. 2002). Although neurons are the main source of the core proteins ensheathing PNNs, astrocytes play an important regulatory role in PNN maturation and maintenance, partly by influencing enzymes like ECM‐remodelling matrix metalloproteinases (MMPs) and have been shown to control the closure of the critical period in both rodents (Min et al. 2024; Ribot et al. 2021) and invertebrates (Ackerman et al. 2021). Thus, our hypothesis is that there is a reciprocal interaction between astrocytes and inhibitory neurons during maturation that ultimately determines the change from a developing to a mature functioning system.

## 6 Astrocyte Factors Affecting Synapse Development, Maturation and Refinement

Astrocytes play a pivotal role in the development and maturation of excitatory synapses in the central nervous system through the release of soluble factors and via direct cell adhesion mechanisms (Table 1). They secrete factors that contribute to excitatory synapse development, including Thrombospondins, which promote silent synapse development (Christopherson et al. 2005; Eroglu et al. 2009); Glypicans (4, 5 and 6), which promote active synapse development (Allen et al. 2012; Bosworth et al. 2025); Hevin and SPARC, which regulate the structural maturation of synapses (Kucukdereli et al. 2011); and Chordin‐like1, which induces the maturation of GluA2‐containing synapses (Blanco‐Suarez et al. 2018). Additionally, astrocytes promote synapse development via cell adhesion molecules. EphrinA3 and EphrinB1, the ligands of the Eph receptor tyrosine kinases, are both negative regulators of neuronal synapses (Murai et al. 2003; Nguyen et al. 2020). Astrocyte neuroligins and their interactions with neuronal neurexins, which are cell adhesion proteins, have also been shown to affect the morphogenesis of astrocytes, with Neuroligin2 specifically shown to affect synapse numbers in the brain (Stogsdill et al. 2017; see Golf et al. 2025 for a contradictory report). Astrocytes also participate in the refinement of synapses through the cell adhesion factor Megf10 and the tyrosine kinase receptor Mertk (Chung et al. 2013).

**TABLE 1:** Astrocyte secreted and contact‐mediated factors associated with excitatory and/or inhibitory synapses.

Synapse type	Factor	Observed function	Origin of factor	References
Excitatory	Thrombospondins	Promotes silent excitatory synapse development	Astrocyte	Christopherson et al. (2005); Eroglu et al. (2009)
	Glypicans (4,5,6)	Promotes active excitatory synapse development	Astrocyte	Allen et al. (2012); Bosworth et al. (2025)
	Chordin‐like 1	Induces maturation of GluA2‐containing excitatory synapses	Astrocyte	Blanco‐Suarez et al. (2018)
Inhibitory	BDNF & NT3	Induces GABAa receptor clustering via neurotrophin signalling	Neuronal	Elmariah et al. (2005)
	Astrocytic EphrinB1	Facilitates PV+ neuron and pyramidal neuron connectivity	Astrocytic membrane‐bound	Sutley‐Koury et al. (2024)
	Neurocan	Regulates inhibitory synaptogenesis; C‐terminal localises to SST+ inhibitory synapses	Astrocyte	Irala et al. (2024)
	NrCAM	Cell adhesion molecule that promotes inhibitory synaptogenesis	Neuron & astrocyte membrane protein	Takano et al. (2020)
Both	Hevin & SPARC	Regulates structural maturation of synapses	Astrocyte	Kucukdereli et al. (2011)
	EphrinA3 & EphrinB1	Negative regulators of synapses	Membrane‐bound, expressed by astrocytes and neurons	Murai et al. (2003); Nguyen et al. (2020)
	Neuroligins & Neurexins	Adhesion molecules; regulates astrocyte morphogenesis and synapse numbers	Neuronal membrane proteins	Stogsdill et al. (2017); Golf et al. (2025)
	Megf10 & Mertk	Receptors for synaptic pruning and refinement	Astrocytic membrane proteins	Chung et al. (2013)
	TGF‐1	Promotes excitatory and inhibitory synaptogenesis	Astrocyte	Diniz et al. (2014)
	Ɣ‐protocadherins	Neuronal adhesion molecules regulating excitatory and inhibitory synapses	Neuronal membrane proteins	Garrett and Weiner (2009)

While excitatory synapse development and maturation have been extensively studied, a few studies have focused on the role of astrocytes in inhibitory synapse formation. Astrocytes promote inhibitory synapse formation and regulate GABAa receptor clustering via neurotrophic BDNF and NT3 signalling (Elmariah et al. 2005). Transforming growth factor beta1 (TGF‐β1), a multifunctional growth factor, was also shown to be an astrocyte‐secreted factor that promotes both excitatory and inhibitory synaptogenesis (Diniz et al. 2014). Astrocytic EphrinB1 was found to facilitate the connectivity between parvalbumin and pyramidal cells in the hippocampus (Sutley‐Koury et al. 2024). In the same year, Neurocan, a chondroitin sulfate proteoglycan secreted by astrocytes, was found to be a key regulator of inhibitory synaptogenesis (Irala et al. 2024). After secretion, Neurocan is proteolytically cleaved, and its C‐terminal fragment specifically localises to synapses, where it is particularly enriched at SST+ inhibitory synapses. Knockout mice without Neurocan or solely its C‐terminal domain show a reduction in both the number and efficiency of inhibitory synapses.

Contact‐mediated interactions with inhibitory synapses were also found. Ɣ‐protocadherins, a family of neuronal adhesion molecules, affect both excitatory and inhibitory synaptogenesis (Garrett and Weiner 2009). More recently, the cell adhesion molecule, NrCAM, was also discovered to promote inhibitory synaptogenesis. When NrCAM was depleted in astrocytes or neurons, a significant decrease in inhibitory synapses was shown (Takano et al. 2020). This suggests that, as in excitatory synapses, there is a combination of astrocyte‐secreted factors and cell adhesion mechanisms that promote the development and maturation of inhibitory synapses. Future studies will need to integrate the above‐identified factors and determine the conditions under which astrocytes utilise each of these pathways.

## 7 Astrocyte Effects on Inhibitory Circuit Maturation

Beyond their role in supporting inhibitory synaptogenesis, astrocytes actively contribute to the maturation of inhibitory neurons, shaping their structural development, functional properties and integration into circuitry. This maturation can be assessed through several features, including morphological complexity, developmental survival, electrophysiological characteristics and association with PNNs. For instance, in vitro studies demonstrated that astrocytes enhance morphological maturation in GABAergic neurons by promoting axon growth and branching (Hughes et al. 2010). in vivo evidence further underscores the importance of astrocytic signalling pathways: Deletion of the fibroblast growth factor receptor Fgfr1 from astrocytes and radial glia led to long‐term reductions in the abundance of specific inhibitory neuron subtypes such as PV+, SST+ and calbindin (CB+) expressing neurons (Smith et al. 2008, 2014). Similarly, selective loss of astrocytic EphrinB1 during early postnatal development resulted in fewer hippocampal PV+ neurons by P28, suggesting impaired maturation (Nguyen et al. 2020). Functional maturation is also impacted by astrocytic factors: knockdown of Connexin 30 in astrocytes reduced both PNN density and the frequency of spontaneous and miniature inhibitory postsynaptic currents (sIPSCs and mIPSCs), highlighting a diminished maturation state of inhibitory circuits (Ribot et al. 2021). Moreover, deletion of the cannabinoid receptor 1 (CB1) from astrocytes, but not from inhibitory neurons themselves, led to more pronounced short‐term depression of inhibitory postsynaptic currents (IPSCs), a hallmark of immature synaptic function (Min et al. 2024).

A recent preprint shows that astrocytic secretion of cellular communication network factor 1 (CCN1) regulates inhibitory circuit maturation in vivo in the visual cortex (Sancho et al. 2024). Expression of CCN1 during the critical period accelerated PV+ neuron maturation, as evidenced by premature PNN formation, while also biasing excitatory synaptogenesis toward PV+ neurons over pyramidal cells. Astrocytes provide some of the crucial ECM proteins that surround PNNs, such as brevican, which is found on PNNs and synapses of basket cells, but not on chandelier cells (Favuzzi et al. 2017; Yamada et al. 1997). When Favuzzi and colleagues removed brevican from basket cells, these cells developed fewer glutamatergic synapses and reduced excitation, with its deletion significantly impairing working and short‐term memory in mice. This was found to be specific to AMPA receptors, and not NMDA or metabotropic receptors. In a recent preprint, Mecp2‐null astrocytes in vitro were found to increase PNN protein HAPLN1 in neurons, a crucial stabilising protein within PNNs that modulates PNN formation (Sinha et al. 2025).

Collectively, these findings indicate that astrocytes can influence multiple aspects of inhibitory neuron maturation through diverse pathways (Table 2). Future studies should examine whether these mechanisms are activity‐dependent and require the coordination of signalling between astrocytes and neurons.

**TABLE 2:** Astrocyte effects on inhibitory neuron maturation.

Astrocytic factor	Condition	Measurement	Inhibitory cell type	Observed effect	Ref.
Unknown	In vitro	Axon length and branching	GABAergic	Increase	Hughes et al. (2010)
Fgfr1	In vivo cKO	Neuron numbers	PV+, SST+, CB+	Decrease	Smith et al. (2008, 2014)
EphrinB1	In vivo cKO	Neuron numbers	Hippocampal PV+	Decrease	Nguyen et al. (2020)
Connexin30	In vivo cKD	PNN density, IPSCs	PNNs, GABAergic	Decrease	Ribot et al. (2021)
CB1 receptor	In vivo cKO	Short‐term depression of IPSCs	GABAergic	Increase	Min et al. (2024)
Brevican	In vivo KO	Glutamatergic synapse/PNN numbers; excitation	PV+ basket cells	Decrease	Favuzzi et al. (2017)
CCN1	In vivo overexpression	PNN density	PV+ and PNNs	Increase	Sancho et al. (2024)
Mecp2	In vitro KO	HAPLN2 protein	Neurons	Increase	Sinha et al. (2025)

Abbreviations: cKO, conditional knockout; KO, full knockout.

## 8 Molecular Mechanisms Enabling Astrocyte–Inhibitory Neuron Crosstalk

If, as we hypothesise, the maturation of inhibitory neurons is activity‐dependent and involves astrocytes, what potential mechanisms could mediate this process?

One option is through neurotrophin signalling. Brain‐derived neurotrophin factor (BDNF) increases expression in the visual cortex of mice after eye opening and is regulated by neuronal activity (Bozzi et al. 1995; Castrén et al. 1992). It has been shown that overexpression of BDNF in pyramidal neurons leads to earlier maturation of inhibitory neurons, a shift in the critical period for vision, and earlier maturation of visual acuity (Huang et al. 1999), while knockout of BDNF resulted in a delay or complete loss of inhibitory neuron maturation (Itami et al. 2007). Within organotypic cultures of visual cortex neurons, parvalbumin expression was accelerated by the addition of BDNF in a Trk‐dependent manner (Patz et al. 2004). in vitro, postnatal astrocytes are able to respond to neuronal BDNF with calcium increases (Jaudon et al. 2021), affecting the internalisation of astrocytic GAT‐1 transporters and promoting GABA uptake (Vaz et al. 2011). Astrocyte maturation is also dependent on BDNF through the binding to TrkB.T1 receptors on astrocytes (Holt et al. 2019). Thus, BDNF could function as a potential mediator between early developmental activity, astrocytes and inhibitory neuron maturation.

Fibroblast growth factors represent another potential avenue, as they have been implicated in regulating inhibitory developmental processes. Recruitment of inhibitory neurons to the visual thalamus depends on retinal inputs mediated by the secreted cell signalling molecule Fgf15. This factor, capable of long‐range action (Ornitz and Itoh 2015) and expressed by a subset of thalamic astrocytes (Su, Charalambakis, et al. 2020), was found to be dependent on sonic‐hedgehog signalling (Somaiya et al. 2022). Since both Fgf expression (Huang et al. 2017) and sonic‐hedgehog release (Su et al. 2017) are induced by neuronal activity, this suggests another potential linking mechanism between astrocyte signalling and inhibitory neuron development and maturation.

MMPs are a family of zinc‐dependent proteolytic enzymes secreted by neurons and glia to remodel the extracellular matrix and directly alter PNNs (see Huntley 2012 for a review). Their release is tightly regulated in an activity‐dependent manner by both neurons and glia, enabling dynamic modulation in the development and later adulthood of brain circuitry (Dziembowska et al. 2012). Among MMPs, MMP‐9 has been extensively studied for its role in a variety of brain disorders ranging from epilepsy, autoimmune disorders and multiple sclerosis (Rempe et al. 2016), but also in development and sensory circuit formation during critical periods (Small and Crawford 2016). Importantly, astrocytic contributions to the closure of critical periods were found to be mediated by the regulation of MMP‐9 (Ribot et al. 2021). Dysfunctional elevations of MMP‐9 have been implicated in excessive breakdown of PNNs and decreased PV+ neuron activity, thus leading to imbalances in E/I, decreased numbers of PNNs, and unsurprisingly, to neurodevelopmental disorders where E/I is imbalanced such as in FXS and autism (Wen, Afroz, et al. 2018). Also, MMP‐1 is notable for its predominant expression by astrocytes leading to the activation of protease‐activated receptor‐1 at inhibitory synapses (Allen et al. 2016; Al‐muhtasib et al. 2018; Wiera and Mozrzymas 2021). This, in turn, leads to the stimulation of Bestrophin‐1, a calcium‐activated anion channel that enables astrocytes to release GABA, thereby further contributing to tonic inhibition of neuronal circuits (Joo et al. 2024). These findings reveal MMPs as key mediators of astrocyte–inhibitory neuron signalling within the ECM, suggesting that other members of the protease family may also play significant roles in this interaction.

We have highlighted three potential mechanisms that may mediate neuronal activity and astrocyte–inhibitory neuron interactions; though further studies are needed to clarify whether these or additional pathways are involved.

## 9 Astrocyte–Inhibitory Neuron Crosstalk—Functional Implications

The interactions between astrocyte and inhibitory neuron maturation could have implications at the level of circuit function, sensory perception and behaviour. A few examples of this have already been described. Manipulation of astrocyte GABAb receptors in developing Drosophila altered levels of the GABA transporter GAT on astrocytes and prevented the hyperexcitability of neurons in a seizure model (Muthukumar et al. 2014). The interactions between astrocytes and SST+ neurons specifically involve GABAb receptors and GAT3‐dependent calcium signalling, which trigger a downstream cascade activating A1 adenosine receptors (Mariotti et al. 2018; Matos et al. 2018). This sequence of events ultimately enhances the inhibitory effect of SST+ neurons on pyramidal neurons. In the adult brain, photostimulation of astrocytes significantly increased PV+ neuron activity while also modestly modulating pyramidal and SST+ neurons (Perea et al. 2014). This astrocyte‐driven modulation subsequently influenced plasticity in the visual cortex, altering orientation preference and spatial frequency tuning in mice. It should be noted, though, that the effects seen could be a result of photostimulation‐driven accumulation of extracellular potassium and not direct astrocyte‐induced activation (Octeau et al. 2019).

The constitutive loss of Fgfr1 from astrocytes and radial glia resulted in hyperactivity in mice that was correlated with the loss of PV+ neurons in the cortex (Smith et al. 2008). When EphrinB1 was deleted from astrocytes, no evidence of hyperactivity was found, but rather impaired social behaviours (Nguyen et al. 2020). In a follow‐up study, the same group found that EphrinB1 in astrocytes affected seizure susceptibility and repetitive behaviours (Sutley‐Koury et al. 2024). These results show that the interaction between astrocytes and inhibitory neurons is important to control the excitability of neurons and ultimately behaviour.

The proper maturation of inhibitory neurons is required for the promotion of plasticity in sensory areas (Fagiolini and Hensch 2000; Kuhlman et al. 2010), and when maturation is altered, this could cause changes to the closure of critical periods of plasticity which would affect sensory processing (Huntley 1997). Rats that were whisker‐trimmed during the critical period of somatosensation were deficient in distinguishing between two types of rough textures even months after the whiskers had regrown (Carvell and Simons 1996) and displayed shorter crossable distances when tested on a gap‐crossing test (Lee et al. 2009). Mice showed similar effects on the gap‐crossing tests as well as deficits in social behaviours (Soumiya et al. 2016). The acceleration of inhibitory neuron maturation by overexpression of BDNF resulted in earlier closure of the critical period for visual plasticity and accelerated acquisition of visual acuity (Huang et al. 1999). In nonsensory areas of the cortex, like the frontal cortex, the prolonged maturation of inhibitory neurons is required to enable goal‐directed decision‐making (Mastro et al. 2025). Therefore, the interactions between astrocytes and inhibitory neurons during development could lead to changes in adult sensory processing, decision‐making and cognition.

## 10 Conclusion, Future Perspectives and Open Questions

Creating a holistic picture of the developmental processes that build the mature brain is not complete without understanding the active role of astrocytes and inhibitory neurons. Here, we have explored the evidence for this, starting with the coincidental emergence of astrocytes with inhibitory neurons, and their reciprocal maturation to become part of the growing network. We discussed the importance of astrocytes as GABAceptive cells and how spontaneous activity of neurons and astrocytes helps further promote morphological complexity and circuit development. The exact role of early astrocyte spontaneous activity is multifaceted, but still underexplored. Our hypothesis is that neuronal and astrocytic coordinated activity interacts and instructs inhibitory neuron maturation through a variety of potential mechanisms that we have highlighted in this review.

The following questions remain:

- Is astrocyte spontaneous activity necessary during development for inhibitory circuit maturation?
- What conditions are required for astrocytes to secrete factors affecting synapse development?
- Is the timing of astrocyte effects on inhibitory and excitatory synapses distinct?
- How are astrocytes involved in the GABA shift?
- Could regional astrocyte heterogeneity explain some of the differences in the timing of onset of mature inhibition in different brain areas?
- How do astrocytes interact with subclasses of inhibitory neurons? Very little is known about their interactions with chandelier cells or with NPY neurons.

Future studies will be necessary to address these open questions. Defining the involvement of astrocytes in the establishment of mature neuronal circuits in the healthy brain is crucial to our understanding of disordered states, as in the case of neurodevelopmental disorders, where there is evidence of excitatory and inhibitory imbalances.

## Author Contributions

**Niina Lehti Tauriala:** conceptualization, visualization, writing – original draft, writing – review and editing. **Vered Kellner:** conceptualization, funding acquisition, project administration, resources, supervision, visualization, writing – original draft, writing – review and editing.

## Conflicts of Interest

The authors declare no conflicts of interest.

## Peer Review

The peer review history for this article is available at https://www.webofscience.com/api/gateway/wos/peer‐review/10.1111/jnc.70266.

## Data Availability

Data sharing is not applicable to this article because no new data were created or analysed in this study.
